# Towards High-Performance Pockels Effect-Based Modulators: Review and Projections

**DOI:** 10.3390/mi15070865

**Published:** 2024-06-30

**Authors:** Yu Li, Muhan Sun, Ting Miao, Jianping Chen

**Affiliations:** 1 State Key Laboratory of Advanced Optical Communication Systems and Networks, Department of Electronic Engineering, Shanghai Jiao Tong University, Shanghai 200240, China; yuli_seiee@sjtu.edu.cn (Y.L.); smh2001@sjtu.edu.cn (M.S.); miaoting@sjtu.edu.cn (T.M.); 2 SJTU-Pinghu Institute of Intelligent Optoelectronics, Pinghu 314200, China

**Keywords:** high-speed modulators, Pockels effect, ferroelectric materials

## Abstract

The ever-increasing demand for high-speed data transmission in telecommunications and data centers has driven the development of advanced on-chip integrated electro-optic modulators. Silicon modulators, constrained by the relatively weak carrier dispersion effect, face challenges in meeting the stringent requirements of next-generation photonic integrated circuits. Consequently, there has been a growing interest in Pockels effect-based electro-optic modulators, leveraging ferroelectric materials like LiNbO_3_, BaTiO_3_, PZT, and LaTiO_3_. Attributed to the large first-order electro-optic coefficient, researchers have delved into developing modulators with expansive bandwidth, low power consumption, compact size, and linear response. This paper reviews the working principles, fabrication techniques, integration schemes, and recent highlights in Pockels effect-based modulators.

## 1. Introduction

Thanks to the development of artificial intelligence, big data, media flow, and other applications, expanding data demands have led to an exponential growth in new hyperscale data centers [1,2,3], resulting in ever-increasing bandwidth requirements for optical transceiver modules. Nowadays, Microsoft, Google, et al., have widely adopted 100 G and partially updated 200 G optical transceiver modules in their data centers. In addition, 400 G [4,5,6] and 800 G [7,8] transceiver modules have already been prototyped and are being produced in small volumes by suppliers, such as Intel [8] and Marvell [3]. Electro-optic (EO) effect-based modulators are commonly employed for facilitating electric-to-optic conversion in an optical transmitter for its fast response time compared to other modulation mechanisms, such as the thermo-optic effect [9,10,11]. Specifically, silicon-based Mach–Zehnder modulators (MZMs) are widely adopted and commercialized in optical transceiver modules. However, the lattice structure of silicon is centrosymmetric and thus has no first-order EO effect (also named the Pockels effect [12]). The modulation in silicon is realized through second-order non-linear free-carrier dispersion (FCD) effect [13]. Due to the use of relatively inexpensive silicon materials, the application of the FCD effect earns the benefits of lower costs and can seamlessly integrate with existing CMOS processes, making it suitable for large-scale production. However, the FCD effect in silicon is relatively weak; typically, a carrier concentration variation of 10^17^ cm^−3^ could only induce a refractive index change of 10^−4^, and this significantly limits further improvement in bandwidth and modulation efficiency in silicon-based EO modulators for beyond 100 G/lane transmission. Up to now, the demonstrated silicon-based modulators with a bandwidth exceeding 60 GHz have involved either (i) co-designing MZM with radio frequency integrated circuit (RFIC) drivers [14] or peripheral resistor–capacitor equalization [15] to generate bandwidth gain; (ii) introducing the slow-light effect through phase-shift grating couplers which sacrificed the driving voltages [16,17]; or (iii) replacing MZM structures with compact microrings [18,19], which requires a complicated wavelength-locking scheme. These methodologies still fundamentally rely on FCD effects; hence, they are unable to completely address the challenges associated with the relationship between carriers and the refractive index.

In order to overcome this issue, ferroelectric-material-based modulators have been studied in recent years. Inspired by the traditional bulk lithium niobate (LiNbO_3_) modulators in fiber communications, integrated thin-film lithium niobate modulators based on the Pockels effect were initially investigated. With an EO coefficient of 30 pm/V [20,21], modulators with a bandwidth exceeding 100 GHz have been demonstrated [22,23,24,25]. This breakthrough has sparked research interests in Pockels effect-based modulators, leading to the successive exploration of other ferroelectric materials with a higher EO coefficient, including barium titanate (BaTiO_3_) [26,27,28], lithium tantalite (LaTiO_3_) [29], lead zirconate titanate (PZT) [30], et al. The primary advantage of the Pockels effect lies in its high electro-optic coefficient, which enables significant modulation depth at low voltages. This characteristic allows ferroelectric-material-based modulators to achieve efficient modulation under low power consumption conditions. Additionally, ferroelectric materials typically exhibit extremely fast response times, reaching picosecond or even sub-picosecond levels, making them suitable for ultra-high-speed optical communication and modulators.

Research on other materials such as phase change material (PCM) [31,32] and indium tin oxide (ITO) [33,34,35] has also been reported; however, they suffered from bandwidth limitations due to a long electro-optic response time and thus are not favored in high-speed modulation. Two-dimensional (2D) material-assisted modulation has also been developed for its ultra-fast response times and compact size [36,37,38]. Specifically, the graphene-assisted electro-absorption (EA)-based modulator has achieved a bandwidth of 39 GHz with a footprint of 27 μm^2^ [36]. However, limited by the weak graphene/dielectric combination and the limited quality of graphene, EA graphene modulators encounter difficulties in achieving high speed and high modulation efficiencies simultaneously. Two-dimensional material-based modulators also face challenges with material integration, stability, and scalability in manufacturing processes.

Considering that the next-generation modulators for photonic transceivers and high-speed computing emphasize large bandwidth, low power consumption, and compact size, this review focuses on Pockels effect-based modulators, which have the potential to achieve a wide bandwidth and high modulation efficiency, as well as compact size. In this review, Section 2 presents the fundamentals of the Pockels effect and the derivation of electro-optic coefficients on various ferroelectric material platforms. Section 3 provides a comprehensive overview of the state of the art in Pockels effect-based modulators. Finally, Section 4 provides conclusions and perspectives.

## 2. Fundamentals of Pockels Effect-Based Modulators

The Pockels effect, also known as the linear EO effect, is a phenomenon in non-centrosymmetric materials where the refractive index changes linearly in response to an applied electric field. This effect was first described by Friedrich Pockels in 1893 [39]. It provides a mechanism for the manipulation of light using electric fields with an ultra-fast response time. Based on a lattice structure and physical properties, typical materials that exhibit the Pockels effect can be classified into four categories: (i) crystal compounds in the form of ABO_3_, such as LiNbO_3_, BaTiO_3_, and LiTaO_3_, and other ferroelectric materials; (ii) compounds with a cubic or hexagonal lattice structure in the form of AB, such as gallium arsenide (GaAs), cadmium telluride (CdTe), and other III-V materials; (iii) the MH_2_XO_4_ crystal family (where M = K, Na, NH_4_^+^; X = As, P), such as potassium dihydrogen phosphate (KH_2_PO_4_), ammonium dihydrogen phosphate (NH_4_H_2_PO_4_), and other inorganic compounds; and (iv) other materials with non-centrosymmetric crystal structures.

**Figure 1:**
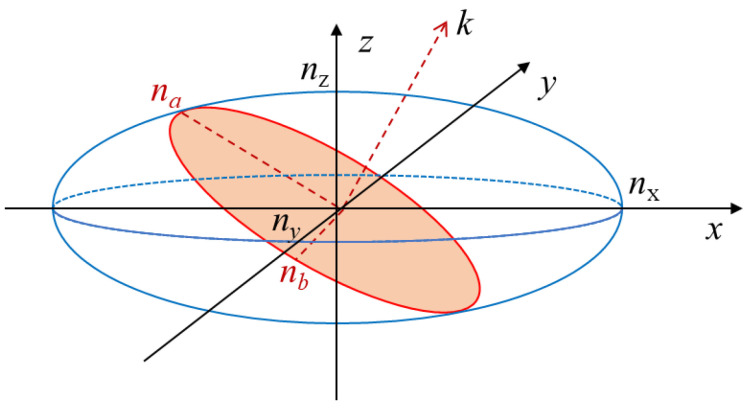
Index ellipsoid of the EO crystal without applied electric field. *k* is the light beam wave vector.

For an anisotropic material, the surface of constant energy density in the field vector space is an ellipsoid in the principal axis coordinate system, as shown in Figure 1, leading to an associated principal refractive index in each (orthogonal) principal direction. The equation for the index ellipse can be expressed as follows:

(1) $${\left(\frac{1}{n^{2}}\right)}_{1}x^{2}+{\left(\frac{1}{n^{2}}\right)}_{2}y^{2}+{\left(\frac{1}{n^{2}}\right)}_{3}z^{2}+2{\left(\frac{1}{n^{2}}\right)}_{4}yz+2{\left(\frac{1}{n^{2}}\right)}_{5}xz+2{\left(\frac{1}{n^{2}}\right)}_{6}xy=1$$

where (1/*n*^2^)*_i_* is the optical indicatrix. Upon the employment of an electric field, the optical indicatrix changes as follows:

(2) $${∆\left(\frac{1}{n^{2}}\right)}_{i}=\sum_{j}r_{ijk}E_{j}$$

where $E_{j}$ is the electric field, and $r_{ijk}$ is the linear EO tensor. Given that an ellipse coordinate system is defined by three axes, the $r_{ijk}$ is a 3 × 3 × 3 matrix with 27 elements. Identical tensors arising from the physical symmetry can be removed to reduce the matrix complexity, leaving 18 independent elements that can be rearranged into a 3 × 6 matrix, as shown below:

(3) $$r_{ij}=\left[\begin{array}{c} \begin{array}{ccc} r_{11} & r_{12} & r_{13}\\ r_{21} & r_{22} & r_{23}\\ r_{31} & r_{32} & r_{33} \end{array}\\ \begin{array}{ccc} r_{41} & r_{42} & r_{43}\\ r_{51} & r_{52} & r_{53}\\ r_{51} & r_{62} & r_{63} \end{array} \end{array}\right]$$

For particular Pockels crystals with specific point group symmetry structures, the number of non-zero tensors could be further reduced.

### 2.1. Pockels Effect for Materials in Point Group 3 m

Point group 3 m [40] is one of the 32 crystallographic point groups, which is characterized by a three-fold rotational axis (120-degree rotation symmetry) along one direction and three mirror planes intersecting this axis. The “3” denotes the three-fold rotational symmetry, and the “m” represents the mirror planes. Using LiNbO_3_ as an example, with its rhombohedral lattice system and trigonal crystal structure, it belongs to point group 3 m. Here, $r_{12}$ = $r_{61}$ = −$r_{22}$, $r_{23}$ = $r_{13}$, $r_{42}$ = $r_{51}$; thus, the tensor matrix could be simplified in the following form:

(4) $$r_{ij}=\left[\begin{array}{ccc} 0 & {-r}_{22} & r_{13}\\ 0 & r_{22} & r_{13}\\ 0 & 0 & r_{33}\\ 0 & r_{42} & 0\\ r_{42} & 0 & 0\\ r_{22} & 0 & 0 \end{array}\right]$$

As shown in Table 1, LiNbO_3_ has a maximum EO coefficient of $r_{33}$. Therefore, aligning the electric field *E* with the Z-axis, assuming light travels in the same direction for waveguide-based modulators, leads to an optimized effective EO coefficient. Then, the corresponding index ellipsoid in the presence of $E_{z}$ could be streamlined to the following:

(5) $$\left(\frac{1}{n_{x}^{2}}+r_{13}E_{z}\right)x^{2}+\left(\frac{1}{n_{y}^{2}}+r_{13}E_{z}\right)y^{2}+\left(\frac{1}{n_{z}^{2}}-r_{33}E_{z}\right)z^{2}=1$$

Notice that for the index ellipse of LiNbO_3_, the refractive index along the x-axis and y-axis is equal, typically denoted as ordinary index $n_{o}$, and the refractive index along the z-axis is extraordinary index $n_{e}$. By considering minor variations in the refractive index upon the applied electric field, the formulations for the refractive index along the three axes can be simplified as follows:

(6) $$n_{x}=n_{y}=n_{o}+\Delta n_{x}\approx n_{o}-\frac{1}{2}n_{o}^{3}r_{13}E_{z}$$

(7) $$n_{z}=n_{e}+\Delta n_{z}\approx n_{e}-\frac{1}{2}n_{e}^{3}r_{33}E_{z}$$

where $\Delta n_{x}$ and $\Delta n_{z}$ are the refractive changes upon the applied electric field $E_{z}$. For waveguide-based MZMs, the refractive index in Equations (6) and (7) needs to be supplanted by the waveguide effective index $n_{eff}$.

Upon a voltage V applied to the MZM waveguide phase shifter, the phase difference ∆∅ along the two arms of the LiNbO_3_ MZM is as follows:

(8) $$∆\emptyset =\frac{2\pi }{\lambda }∆n_{eff}L$$

where L is the length of the phase shifter in MZM, and λ is the input wavelength. Furthermore, taking into consideration the overlapping factor Γ between the electric field and the optical mode in the waveguide, ∆∅ could be expressed using the effective EO coefficient r and $E_{z}$ as follows:

(9) $$∆\emptyset =\Gamma \frac{\pi Vn_{eff}^{3}rL}{d\lambda }$$

where d is the distance between the parallel electrodes. The modulation efficiency of the MZM is reflected by the half-wave voltage–length product:

(10) $$V_{\pi }L=\frac{d\lambda }{n_{eff}^{3}r\Gamma }$$

For the LiNbO_3_-based MZM, r is related to $r_{33}$. From the equation, we can see that for a given material, the way to increase the modulation efficiency is either by varying the $n_{eff}$ through waveguide dimension optimization or by enhancing the overlapping factor through electrode design.

### 2.2. Pockels Effect for Materials in Point Group 4 mm

An alternative method to enhance the modulation efficiency is to use other ferroelectric materials, such as LiTaO_3_, which is in the same point group as LiNbO_3_. However, for materials with a lattice structure in a distinct point group, the effective EO tensor matrix is varied. For BaTiO_3_, above its Curie temperature of 120 °C, the lattice structure is in cubic phase with a point group of m3m. In this phase, the BaTiO_3_ enters paraelectric mode and is centrosymmetric. At room temperature, the crystal structure is in tetragonal phase with a point group of 4 mm. Thus, this review mainly focuses on the tetragonal phase. Specifically, point group 4 mm features a four-fold rotational axis along the principal axis with 90-degree rotational symmetry [48]. The first m represents mirror planes that are repeated by the rotational symmetry, and the second m denotes mirror planes that bisect the first set. The tensor matrix for the 4 mm point group could be streamlined in the following form:

(11) $$r_{ij}=\left[\begin{array}{ccc} 0 & 0 & r_{13}\\ 0 & 0 & r_{13}\\ 0 & 0 & r_{33}\\ 0 & r_{51} & 0\\ r_{51} & 0 & 0\\ r_{22} & 0 & 0 \end{array}\right]$$

The corresponding index ellipse is then simplified as follows:

(12) $$\left(\frac{1}{n_{o}^{2}}+r_{13}E_{z}\right)x^{2}+\left(\frac{1}{n_{o}^{2}}+r_{13}E_{z}\right)y^{2}+\left(\frac{1}{n_{e}^{2}}+r_{33}E_{z}\right)z^{2}+\left(r_{51}E_{y}\right)2yz+\left(r_{51}E_{x}\right)2zx=1$$

BaTiO_3_-based modulators could either work in the elongated axis, the c-axis (dominated by $r_{51}$), or in the other two axes (dominated by the combined effect of $r_{51}$, $r_{13}$, and $r_{33}$). In the c-axis configuration, the optic axis is perpendicular to the BaTiO_3_ thin film. The resulting waveguide $n_{eff}$ change correlates with the square of $E_{x}$, which requires a relatively large bias voltage and is not favored for low-power consumption applications.

**Figure 2:**
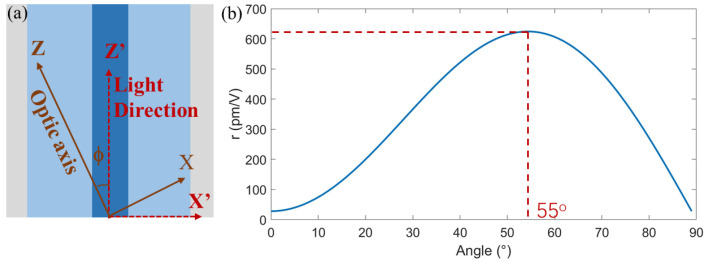
(**a**) Schematic of effective EO coefficient module in an a-axis-oriented BaTiO_3_ platform. (**b**) Effective EO coefficient as a function of tilted angle *ϕ*.

In the a-axis scenario, the optic axis lies within the plane of the BaTiO_3_ thin film, allowing for the alignment of the waveguide either parallel to the optic axis or at a specified angle, as shown in Figure 2a. Meanwhile, only the TE mode can be modulated in the a-axis scenario. The equivalent refractive index $n_{TE}$ and EO coefficient $r_{TE}$ for the TE mode of the waveguide, which is tilted at a *ϕ*-degree to the optic axis, can then be expressed as follows:

(13) $${n_{eff}=n}_{TE}=n_{z}^{\prime }=\frac{n_{o}n_{e}}{\sqrt{n_{e}^{2}\sin^{2}\phi +n_{o}^{2}\cos^{2}\phi }}$$

(14) $${r=r}_{TE}=r_{33}\cos^{3}\phi +(r_{13}+2r_{51})\sin^{2}\phi \cos\phi$$

Calculated using the EO coefficients in Table 1, the equivalent EO coefficient as a function of the tilted angle ϕ is shown in Figure 2b. When ϕ = 55°, the highest equivalent $r_{TE}$ = 624 pm/V is obtained with an equivalent $n_{TE}$ of 2.40. Employing the same analysis as in Section 2.1, the modulation efficiency for BaTiO_3_-based MZM can be calculated.

Another Pockels effect material showcased for EO modulation is PZT, an inorganic compound denoted by the chemical formula PbZr_x_Ti_1−x_O_3_. The crystal structure of the traditional PZT is similar to BaTiO_3_, featuring a lower EO coefficient and a higher Curie temperature, as outlined in Table 1. Consequently, the aforementioned analysis is also applicable to the case of PZT.

## 3. Ferroelectric-Material-Based Modulators

### 3.1. LiNbO_3_-Based Modulators

Leveraging the relatively mature crystal fabrication industry and its traditional applications in fiber optics, LiNbO_3_ stands out as the most prevalent and extensively researched material for Pockels effect-based integrated modulators. Traditional bulk LiNbO_3_ EO modulators, based on proton exchange or titanium diffusion, have a low refractive index contrast between the waveguide and the cladding, leading to a large optical mode. This requires large spacing between the modulation electrodes to avoid optical crosstalk and signal degradation, which results in high driving voltages and large power consumption. To address these limitations, recent exploration of an integrated LiNbO_3_ modulator has employed new fabrication techniques, including hybrid integration and lithium niobate on insulator (LNOI), to realize high-index-contrast LiNbO_3_ waveguides.

**Figure 3:**
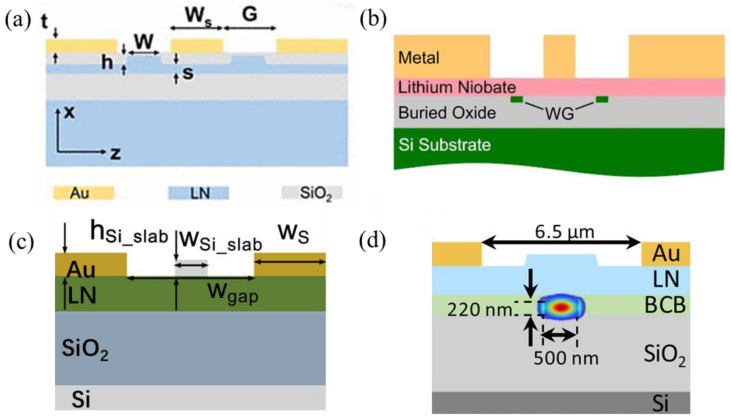
Integration schemes of LiNbO_3_-based modulators. (**a**) LNOI with etched LiNbO_3_ waveguides [49]; (**b**) LiNbO_3_ thin film bonded to SOI wafers [50]; (**c**) Si-loaded LiNbO_3_ thin films [51]; (**d**) LiNbO_3_ thin film bonded to SOI wafers using BCB [52].

The key step towards high-index-contrast LiNbO_3_ waveguides is to produce single-crystalline LiNbO_3_ thin films on low-index dielectric substrates. Over the years, numerous techniques, such as molecular beam epitaxy (MBE), pulsed laser deposition (PLD), and chemical vapor deposition (CVD), have been investigated for the growth of LiNbO_3_ thin films. However, none of these have demonstrated mature single-crystalline LiNbO_3_-thin-film growth capability on low-refractive-index substrates. Thus, nowadays, a more widely used technique is the wafer bonding technique, either by bonding the LiNbO_3_ thin film to a substrate, where the thin film is directly obtained from the bulk LiNbO_3_, or by bonding Si/SiN device layers to LiNbO_3_ substrates. Figure 3 summarizes the commonly used integration schemes of LiNbO_3_-based modulators.

#### 3.1.1. LNOI-Based Modulators

The most adopted approach to obtaining thin-film LiNbO_3_ on low-refractive-index materials is using the smart-cut technique. This technique, analogous to silicon-on-insulator (SOI) fabrication [53], involves the He ion implantation of an LiNbO_3_ wafer, followed by flip-wafer bonding to a carrier wafer. Both the carrier wafer and the bonding wafer are oxidized to enable atomic bonding. Subsequently, the bonded wafer is annealed in order to activate micro-explosions of the implanted ions, causing the LiNbO_3_ wafer to split and leaving a thin film on the carrier wafer. This bonded wafer is then polished and annealed to repair ion-implantation-induced damages. The method was first developed back in the early 2000s at a die scale. Recently, 8-inch LNOI wafers have been commercialized, underscoring the potential for cost-effective LNOI-based modulators and revitalizing the development of LNOI-based devices and systems. Instead of atomic oxide bonding, some researchers [54,55] chose to use polymer adhesive, namely benzocyclobutene (BCB), to bond the wafers due to its low-temperature operation and versatility. However, the limited temperature tolerance of the bonded wafer prevents the employment of high-temperature annealing processes for repairing crystal lattice damages, resulting in high waveguide loss and a low EO coefficient.

One of the challenges for LNOI technology is to achieve effective mode confinement in waveguides. Lithium niobate is relatively chemically inert, making it difficult to precisely etch LiNbO_3_ waveguide profiles with low roughness. Various techniques, including both wet etching and dry etching, have been explored to reduce the waveguide propagation loss.

Wet etching is one of the techniques that were first developed for LiNbO_3_ waveguide formation. Back in 2007, H. Hu et al. [56] demonstrated a 6.5 μm width and 8 μm height waveguide with a TE transmission loss of 0.3 dB/cm. The etching solution was composed of hydrofluoric acid (HF) and nitric acid (HNO_3_), with the addition of ethanol for surface smoothness. Over time, researchers have focused on enhancing etching capabilities through solvent refinement. As of 2023, significant progress has been made by R. Zhuang et al. [57], with a propagation loss down to 0.04 dB/cm for high-quality factor micro-resonators. This wet etching method comprised a solvent of mixed H_2_O_2_ and NH_4_OH at an escalated temperature of 85 °C, followed by 2 h of annealing at 250 °C in order to repair the potential damage induced by ion slicing and wet etching.

Alternatively, leveraging the advantages of high precision, anisotropy, and smooth sidewalls, dry etching using inductively coupled plasma (ICP) reactive-ion etching [58,59,60] is extensively preferred. The fluorine-gas-based ICP etch has the benefit of a fast etching speed but results in the formation of the byproduct LiF, which accumulates on sidewalls and reduces the slope angle. To mitigate the problem, I. Krasnokutska et al. [59] mixed Ar ions with the etching gas, achieving a propagation loss of 0.4 dB/cm and an enhanced slope angle of 75°. Chlorine-based gases, such Cl_2_ and BCl_3_, are also used for etching LiNbO_3_. Compared to fluorine gases, chlorine-based etching produces the byproduct LiCl, which has a lower melting point and thus is able to achieve an improved sidewall angle. M. Bahadori et al. [61] reported fully etched LiNbO_3_ waveguides with a height of 560 nm, using a mixture of Cl_2_, BCl_3_, and Ar, achieving a sidewall angle of 83°. Additionally, C. Shen et al. [62] employed a SiO_2_ mask and a mixture of Ar, Cl_2_, and BCl_3_ as the etching gas, obtaining an etching sidewall angle of close to 80°. There is also research focused on Ar plasma-based physical etching. F. Kaufmann et al. [63] conducted ICP etching using Ar ions alone. When the chamber pressure was reduced from 5 mTorr to 1 mTorr under a 600 V DC bias, the sidewall angle increased from ~52° to 62°, and the optimized propagation loss was 1.55 dB/cm. Furthermore, G. Chen et al. [58] achieved a propagation loss of 0.15 dB/cm for LiNbO_3_ waveguides with a thin-film thickness of 400 nm and a ridge height of 200 nm.

Thanks to the development of LNOI wafers and etching technology, over the past five years, there have been significant advancements in the development of LNOI-based modulators. These devices have seen improvements in both bandwidth and modulation efficiencies and integration with other photonic components. However, there remains a trade-off between the bandwidth and modulation efficiency in LiNbO_3_ modulators due to the intrinsic EO coefficient of the material. Increasing the bandwidth requires minimizing the overlap between the optic and electric fields in order to reduce the capacitance, which consequently lowers the modulation efficiency.

**Figure 4:**
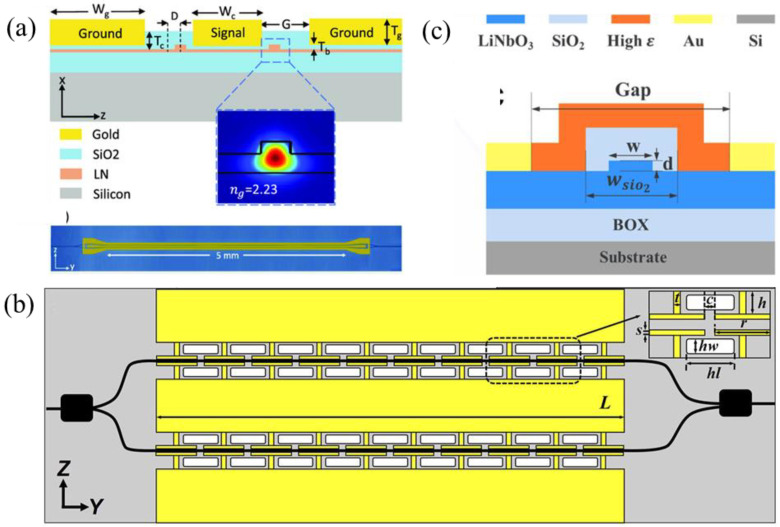
Recent developments in LNOI-based modulators. (**a**) Schematic of an asymmetric waveguides-to-RF pads design [64]; (**b**) top-view schematic of the periodic capacitively loaded traveling-wave electrode design [58]; (**c**) schematic of the waveguide cross-section with isolated trenches and a high-refractive-index cladding [65] (adapted from [65] with permission: Copyright © 2024 Wiley-VCH GmbH).

In order to mitigate these constraints, F. Juneghani et al. [64] achieved an extrapolated bandwidth of 170 GHz with a modulation efficiency of 3.3 V·cm by positioning the optical waveguides asymmetrically relative to the metal electrodes and introducing a thin SiO_2_ dielectric buffer layer beneath the electrodes. As shown in Figure 4a, the asymmetrical waveguide design allowed a strong electric field near the signal electrode while maintaining the distance between the electrodes. The buffer layer mitigated the optical loss and reduced the RF effective index for velocity matching. G. Chen et al. [58] integrated a periodic capacitively loaded travelling-wave electrode to reduce the RF loss and an undercut in the silicon substrate to decrease the RF refractive index, attaining a bandwidth beyond 67 GHz and a tuning efficiency of 2.2 V·cm, as shown in Figure 4b. Alternately, N. Chen et al. [60] enhanced the overlap between the optic and electric fields through introducing isolation trenches around the waveguide to enlarge the refractive index contrast. They further improved the mode confinement through implementing high-refractive-index material as the waveguide cladding [65], achieving a modulation efficiency of 1.4 V·cm and a bandwidth of over 67 GHz, as shown in Figure 4c.

**Figure 5:**
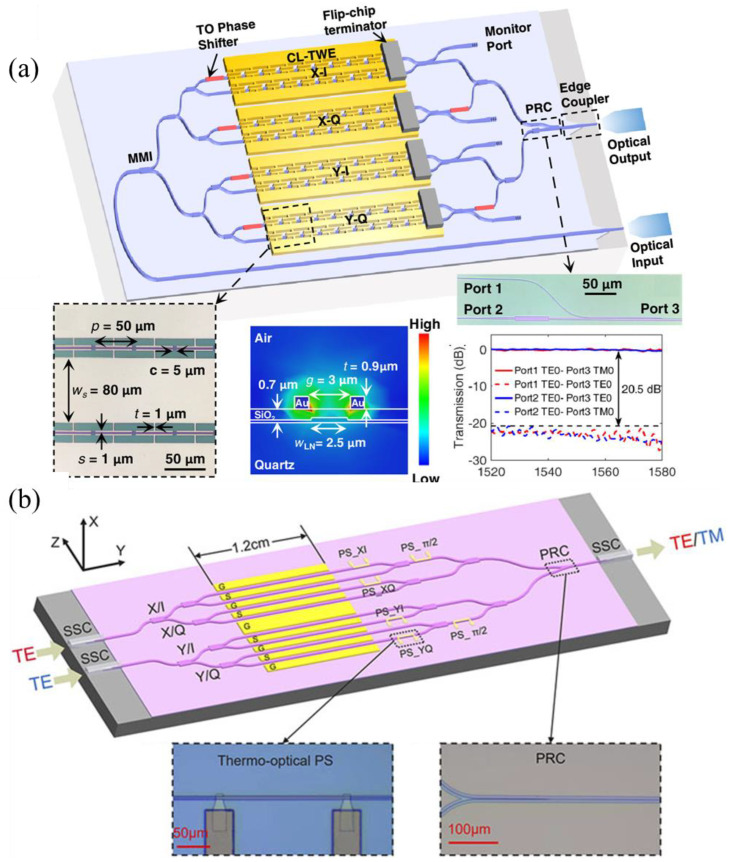
(**a**) A 110-GHz bandwidth dual-polarization IQ modulator supporting 1.96 Tb/s data transmission [23]. (**b**) A dual-polarization IQ modulator supporting 1.6 Tb/s data transmission under 256 QAM [66].

On the other hand, advanced modulation formats [23,66,67] have been employed to further increase the data transmission capability of the modulators. F. Yang et al. [67] achieved a 250 Gb/s data transmission upon six-level pulse amplitude modulation (PAM) using an LNOI-based modulator with a bandwidth of 110 GHz. M. Xu et al. [23] demonstrated a high-performance dual-polarization in-phase quadrature (IQ) modulator for coherent transmission, enabling a single-wavelength 1.96 Tb/s data transmission, as shown in Figure 5a. X. Wang et al. [66] demonstrated a dual-polarization IQ modulator supporting 1.6 Tb/s data transmission under 256 quadrature amplitude modulation (QAM), as shown in Figure 5b.

#### 3.1.2. Modulators with Hybrid-Integrated LiNbO_3_ on Si/SiN Platforms

**Figure 6:**
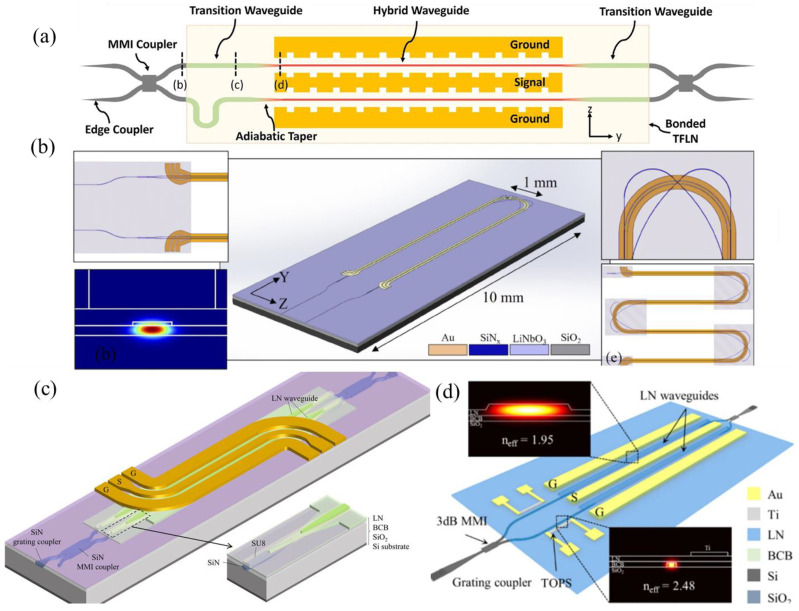
Recent developments in modulators based on LiNbO_3_-Si/SiN hybrid integration schemes. (**a**) Schematic of a LiNbO_3_-on-Si MZM with slow-wave electrodes [73]. (**b**) Schematic of a folded MZM based on LiNbO3-on-SiN platform [75]. (**c**) Schematic of an MZM with dual-tapers design on a BCB-assisted LiNbO_3_-on-SiN platform [68] (adapted from [68] with permission: Copyright © 2024 Wiley-VCH GmbH). (**d**) Schematic of a bias-drift-free BCB-assisted LiNbO_3_-on-Si MZM [76].

To avoid the complexity associated with etching LiNbO_3_ for LNOI-based modulators, an alternative approach involves bonding Si or SiN [68,69] device layers to LiNbO_3_ substrates. In this approach, LiNbO_3_ functions as the slab, and together with the bonded Si or SiN waveguide layers, hybrid Si/SiN-LiNbO_3_ ridge waveguides are created. Through the optimization of the Si/SiN waveguide dimensions and LiNbO_3_ film thickness, the majority of light can be confined in the LiNbO_3_ film. Additionally, the hybrid integration scheme presents an easy and scalable way to integrate modulators with other maturely developed passive components on the Si/SiN platform. Figure 6 depicts the recent development in modulators based on LiNbO_3_-Si/SiN hybrid integration schemes.

The bonding of LiNbO_3_ to Si or SiN layers can be realized at the wafer-to-wafer [70,71,72], die-to-wafer [50,68], or die-to-die [73,74] level. The mainstream bonding technique is hydrophilic bonding. The process begins with surface cleaning of both the LiNbO_3_ and Si/SiN substrates using solvents such as acetone and isopropanol, followed by a rinse in deionized water. Surface activation is then carried out, typically through oxygen plasma treatment, to enhance surface energy and promote strong bonding. In direct bonding, the activated surfaces are then brought into contact under pressure for uniform adhesion. Subsequently, thermal annealing is conducted to enhance the bond by promoting atomic interdiffusion. In 2023, M. Churaev et al. [72] successfully bonded a 4-inch LiNbO_3_ wafer on a 100 mm diameter silicon wafer with Si_3_N_4_ waveguides on top and SiO_2_ as the bonding interface. S. Ghosh et al. [71] demonstrated an Al_2_O_3_-assisted bonding procedure for 100 mm LiNbO_3_ wafers on 200 mm Si_3_N_4_-integrated photonic integrated circuit (PIC) wafers.

Leveraging the bonding technology, P. Weigel et al. [22] demonstrated a 5 mm long hybrid-integrated MZM with a bandwidth of 106 GHz and a V_π_L product of 6.7 V·cm, with 81% light confined in the LiNbO_3_ layer, achieving a large effective EO coefficient and 5% light in the Si waveguide. Furthermore, the same research group reduced the V_π_L product to 3.1 V·cm [73] through the implementation of slow-wave electrodes for improved velocity matching.

Besides LiNbO_3_-Si hybrid integration, the LiNbO_3_-on-SiN platform has garnered research interest due to its wide transmission window, low propagation loss, and cost-effectiveness. A. Ahmed et al. [77] demonstrated a hybrid LiNbO_3_-SiN MZM with a V_π_ below 1 V by extending the EO interaction region to 2.4 cm and reducing the electrode gap between the ground and signal. Taking advantage of a Michelson interferometer (MI) with a doubled EO interaction length, X. Huang et al. [78] achieved exceptional modulation efficiency with a low V_π_L of 1.06 V·cm for a 0.6 mm long MI modulator (MIM). However, the counterpropagation of light in MIMs has limited the device bandwidth to 40 GHz. Alternately, S. Nelan et al. [75] increased the EO interaction length from 6 mm (physical) to 10 mm (effective) by employing a folded waveguide structure. A 180° bend was introduced to overcome the polarity inverse between the optic mode and electric field. This design realized a modulation efficiency of 4 V·cm and a bandwidth of 37.5 GHz.

On the contrary, there is also research on Si [51] or SiN [79] layers bonded to LiNbO_3_ substrates. P. Zhang et al. [79] demonstrated an EO MZM on a SiN-loaded LNOI platform with a coplanar waveguide design for push–pull modulation. The achieved bandwidth was 30 GHz, and the V_π_L was 2.24 V·cm.

In addition to hydrophilic bonding, BCB-assisted LiNbO_3_ hybrid-integrated modulators have also been investigated [25,54,76] due to their ease of fabrication and flexibility in device design. X. Liu et al. [54] proposed a 10 mm long modulator with an estimated bandwidth beyond 300 GHz and a modulation efficiency of 1.2 V·cm. The LiNbO_3_ thin film was bonded to a quartz substrate using BCB, with air-filled undercut regions to enhance the waveguide mode confinement. M. He et al. [25] designed a Si-LiNbO_3_ double-waveguide-layer structure through BCB adhesive die-to-wafer bonding and LiNbO_3_ dry etching, in order to mitigate the constraints of LNOI-only structures and maintain a great optical mode confinement at the same time. Under a single-drive push–pull operation, an EO bandwidth of 70 GHz and a modulation efficiency of 2.2 V·cm was achieved for a 3 mm length modulator.

#### 3.1.3. Summary of LiNbO_3_-Based Modulators

In conclusion, LiNbO_3_-based modulators have undergone rapid development, with various integration methods and fabrication technologies emerging. In order to improve key performance metrics including bandwidth, modulation efficiency, and insertion loss, studies have focused on novel designs, such as electrode engineering, hybrid waveguide configurations, and increasing interaction length schemes. Table 2 summarizes the state of the art of LiNbO_3_-based modulators in the last five years.

**Table 2:** State of the art of LiNbO_3_-based modulators in the last five years.

Year	Scheme	Structure	Length (mm)	V_π_ (V)	V_π_L (V·cm)	Bandwidth (GHz)	Insertion Loss (dB)	Ref.
2023	LNOI	MZM	5	6.6	3.3	170	NA *	[64]
2023	LNOI	MZM	4	3.52	1.41	>67	0.5	[65]
2023	LNOI	MZM	4	3	1.2	>40	2.43	[60]
2022	LNOI	MZM	5	4.74	2.37	>110	NA	[67]
2021	LNOI	MZM	5	3.5	1.75	>40	NA	[80]
2021	LNOI	MZM	13	2.36	3.068	60	2	[81]
2021	LNOI	MZM	4	1.6	0.64	>3	NA	[82]
2020	LNOI	IQM	13	1.9	2.4	>48	1.8	[83]
2023	SiN + LN	MZM	7	4.3	3	37	1	[68]
2022	Si + LN	MZM	5	NA	3.1	110	1.8	[73]
2022	Si + LN	MZM	10	2.2	2.2	>67	0.2	[58]
2022	SiN + LN	MZM	6	4	4	37.5	NA	[75]
2021	SiN + LN	MZM	7.8	2.8	2.18	30	NA	[79]
2021	SiN + LN	MIM	NA	17.8	1.06	>40	NA	[78]
2021	TFLN	MZM	10	NA	1.2	>300	<1	[54]
2020	SiN + LN	MZM	24	0.875	2.11	NA	5.4	[77]

* NA: Not available.

### 3.2. BaTiO_3_-Based Modulators

Recently, BaTiO_3_ emerged as a highly promising material for EO modulators in integrated photonics due to its high EO coefficient. As listed in Table 1, the reported EO coefficient for BaTiO_3_ exceeds 800 pm/V, positioning it as a strong candidate for realizing high-performance modulators.

#### 3.2.1. Heterogenous Growth of BaTiO_3_ Thin Films

The integration of BaTiO_3_ on PICs typically involves the monolithic growth of BaTiO_3_ thin films on substrates. The heterogeneous growth of BaTiO_3_ thin films on substrates has been developed through diverse techniques, including metal–organic chemical vapor deposition (MOCVD) [84], PLD [85,86,87], MBE [88,89], and RF sputtering [90,91]. The thin film is typically grown on magnesium oxide (MgO) substrate or silicon substrate.

Using MgO as a substrate for BaTiO_3_-thin-film growth has been well studied due to its similar lattice structure to BaTiO_3_ and its broad spectral transparency [26,92]. MgO with a cubic lattice structure has a larger lattice constant (4.21 Å) compared to tetragonal-phase BaTiO_3_, causing a lattice mismatch of 4–5% (depending on the axis) [93]. The resulting BaTiO_3_ thin film could be either c-oriented or a-oriented, depending on the deposition methodology, growth conditions, and buffer layers.

Silicon is another commonly used substrate, considering its widespread availability and compatibility with semiconductor manufacturing techniques [94,95,96,97]. However, the lattice constant of silicon is 5.43 Å, leading to a severe lattice mismatch with BaTiO_3_. To address this issue, a buffer layer of SrTiO_3_ is implemented, with a thickness of a few nanometers. The SrTiO_3_ buffer layer helps to alleviate the strain caused by the lattice mismatch between the silicon substrate and the BaTiO_3_ thin film, facilitating the growth of high-quality epitaxial films.

In addition, chemical solution deposition (CDS) using La_2_O_2_CO_3_ template film has also been developed for BaTiO_3_-thin-film growth. E. Picavet et al. [98] successfully fabricated a BaTiO_3_ film of 190 nm thickness. The thin layer featured a c-axis aligned in plane and a Pockels coefficient of 139 pm/V.

#### 3.2.2. State of the Art of BaTiO_3_-Based Modulators

**Figure 7:**
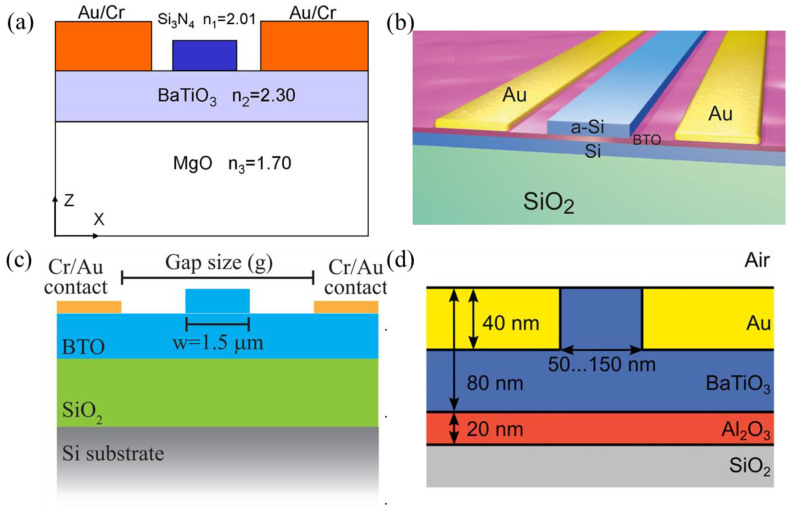
Typical integration schemes of BaTiO_3_-based modulators. (**a**) Cross-sectional schematic of a BaTiO_3_ thin film grown on a MgO substrate, with a deposited SiN layer to form hybrid waveguides [92]. (**b**) A BaTiO_3_ thin film grown on an SOI wafer through a SiTrO_3_ buffer layer, with a deposited amorphous-Si layer to form hybrid waveguides [97] (adapted from [97] with permission: Copyright © 2024, American Chemical Society). (**c**) A BaTiO_3_ thin film grown on an SOI wafer, followed by Ar ion milling to form waveguides [91] (adapted from [91] with permission: Copyright © 2024, American Chemical Society). (**d**) A BaTiO_3_ thin film bonded to a PIC chip [99].

In this section, the state of the art of BaTiO_3_-based modulators is presented. Compared to that of LiNbO_3_ material, the development of BaTiO_3_-based modulators is still in an early stage. Modulators in various configurations and integration schemes have been explored. Figure 7 summarizes the typical integration schemes of BaTiO_3_-based modulators.

Notice that BaTiO_3_ also exhibits chemical inertness. In heterogeneous integration approaches, typically, waveguide formations need to rely on either depositing amorphous-Si or SiN device layers or bonding to additional PIC chips. The nanoscale gap between the silicon or MgO substrate and BaTiO_3_ influences the distribution of the optical field within the BaTiO_3_ layer, requiring precise device design to attain optimal modulation efficiency.

**Figure 8:**
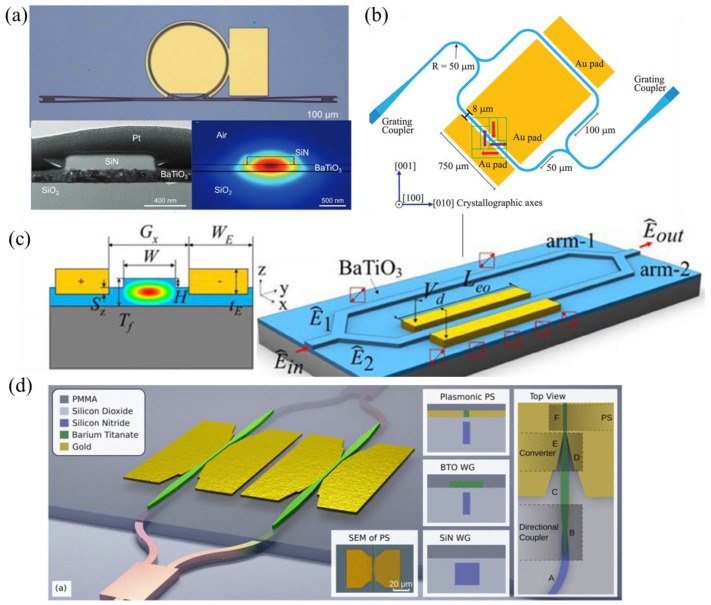
(**a**) A c-axis-oriented microring modulator with a bandwidth of over 24 GHz [98] (adapted from [98] with permission: Copyright © 2024 Wiley-VCH GmbH). (**b**) Schematic of an MZM based on etched BaTiO_3_ waveguides with effective EO coefficient of 89 pm/V [91] (adapted from [91] with permission: Copyright © 2024, American Chemical Society). (**c**) A c-axis-oriented etched BaTiO_3_-based phase-polarization modulation scheme [100] (adapted from [100] with permission: Copyright © 2024 Wiley-VCH GmbH). (**d**) A plasmonic modulator based on the BaTiO_3_-SiN bonding technique [101].

A. Posadas et al. [90] reported an a-axis BaTiO_3_-based MZM with an effective EO coefficient of 157 pm/V and a V_π_L of 0.42 V·cm. The BaTiO_3_ thin film was deposited on SOI wafers using RF sputtering, followed by a polycrystalline silicon-rich SiN layer deposition to form the hybrid waveguides. Instead of SiN, C. Xiong et al. [97] formed the hybrid waveguide using a deposited amorphous-Si layer, with crystalline BaTiO_3_ thin film directly epitaxial on the SOI, achieving an effective Pockels coefficient of 213 pm/V, as shown in Figure 8a. P. Girouard et al. [26] demonstrated a photonic crystal-based modulator with a device length of 1 mm. The measured EO bandwidth was over 40 GHz, and the V_π_L was ~0.66 V·cm.

Another method to create waveguide morphology is through an ion-milling dry-etch technique [91,100], as shown in Figure 8b,c. Z. Dong et al. [91] achieved an etching depth of 175 nm using Ar ions to physically bombard the BaTiO_3_ layer. With an RF-sputtered BaTiO_3_ thin film on the SOI wafer and fully etched BaTiO_3_ waveguides, an MZM with an effective EO coefficient of 89 pm/V was demonstrated, with a corresponding V_π_L of 2.3 V·cm.

In order to use crystalline Si waveguides for potential low propagation loss, F. Eltes et al. [102] proposed to flip-chip bond the BaTiO_3_ films that were grown on SOI substrate to another PIC chip. This process allowed the exposure of the Si device layer of SOI after removing the Si and SiO_2_ layers beneath and thus enabled waveguide patterning on the Si device layer. An MZM supporting a 25 Gb/s data transmission was demonstrated with a waveguide propagation loss of 3 dB/cm and a low V_π_L of 0.2 V·cm.

Alternatively, the heterogeneous-grown BaTiO_3_ thin films could be integrated with Si or SiN device layers using a bonding technique. Lumiphase, for instance, developed a hybrid bonding platform in 2022 [103] in order to attach BaTiO_3_ thin films on damascene SiN waveguides. Leveraging this platform, they reported a series of works on various modulator schemes. A microring modulator with a low propagation loss of 0.7 dB/cm and a V_π_L of below 0.56 V·cm was demonstrated to validate the potential of the platform. In 2023, they reported a BaTiO_3_-based MZM with a footprint of 0.4 mm × 1.6 mm [27]. The device featured an insertion loss of 2 dB and a V_π_L of 4.8 V·mm, with an operating data rate up to 200 Gb/s under linear receiver equalization. Their recent work also involved plasmonic modulators [101,104], as shown in Figure 8d. The light was coupled from SiN waveguides to the plasmonic section through two transition tapers, functioning to realize light transition between SiN-to-BaTiO_3_ and BaTiO_3_-to-plasmonic mode. A modulator with a V_π_L of below 0.1 V·mm, a total insertion loss of 20.3 dB, and a bandwidth of 110 GHz was demonstrated with measured eye diagrams up to 256 GBd. Table 3 summarizes the recent state of the art of BaTiO_3_-integrated modulators.

### 3.3. Pockels Effect Modulators Based on Other Ferroelectric Materials

At present, Pockels effect-based modulators predominantly rely on LiNbO_3_ and BaTiO_3_. However, there are other ferroelectric materials worth noticing that have already shown high-performance capability, such as LiTaO_3_ and PZT.

PZT material has been explored for high-performance modulators for its low cost, lower phase transition temperature, and high EO coefficient. In 2021, D. Ban et al. [108] demonstrated PZT-thin-film growth on sapphire substrates using chemical solution deposition, showcasing an EO coefficient of 133 pm/V. The resulting MZM achieved a V_π_L of 3.6 V at 1550 nm and a verified modulation performance within the 6–12 GHz signal frequency range. In 2022, they further presented a modulator with PZT thin films grown on SiO_2_/Si substrates, featuring an enhanced V_π_L of 1.4 V·cm, a propagation loss of 1.8 dB/cm, and a bandwidth of 12 GHz [109]. Recently, S. Yokoyama et al. [110] demonstrated an MZM supporting 200 Gb/s PAM4 transmission based on PZT and lead lanthanum zirconate titanate (PLZT)-on-insulator platforms. By adding lanthanum to PZT to form PLZT, the EO coefficient is increased. The PZT/PLZT ridge waveguide had a ridge height of 100 nm and width of 1.6 μm; the estimated effective EO coefficient was 86 pm/V and 198 pm/V for PZT and PLZT modulators, respectively. The corresponding V_π_L for the 4 mm long MZM was 1.3 V·cm and 0.88 V·cm at 1550 nm.

**Figure 9:**
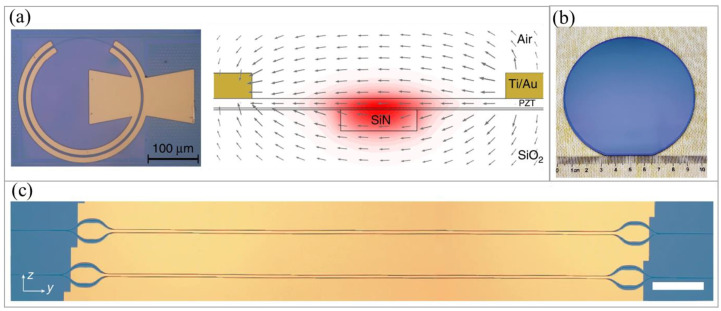
(**a**) Microscope image of a PZT-based microring modulator [30]. (**b**) Photograph of a fabricated 4-inch LTOI wafer [47]. (**c**) Microscope image of a fabricated MZM on the LTOI platform [47].

LiTaO_3_ on insulator (LTOI) has already been commercialized in 5G radio frequency devices, making it a mature platform for integration applications. Compared to LNOI, LTOI exhibits a similar EO coefficient but has an optical birefringence that is over one order of magnitude lower. This is specifically helpful towards realizing waveguide bends with a reduced radius. For a proof-of-concept demonstration, J. Shen et al. [29] reported a microring modulator on an LTOI platform, using a deposited amorphous-Si layer for waveguide profiling. The achieved wavelength tuning efficiency was 12.8 pm/V with a bandwidth of over 20 GHz. Earlier this year, C. Wang et al. [47] achieved an MZM with a V_π_L of 1.9 V·cm and a bandwidth of 40 GHz on a 4-inch LTOI wafer, as shown in Figure 9b,c. The wafer was manufactured using the smart-cut technique, followed by a stepper-based wafer-scale etching process to create low-loss waveguides.

### 3.4. Challenges and Comparison between Ferroelectric-Material-Based Platforms

Leveraging the Pockels effect, ferroelectric-material-based modulators exhibit the capability of achieving high modulation efficiency. Among these materials, BaTiO_3_ exhibits the highest EO coefficient, typically achieving a V_π_L below 0.5 pm/V for EO modulators. On the contrary, both LiNbO_3_ and LaTiO_3_ have relatively lower EO coefficients, requiring specific waveguides or electrode design to lower the V_π_L. Consequently, modulators based on these two materials typically require a phase-shifter length exceeding 5 mm. The optical birefringence of LaTiO_3_ is an order of magnitude lower than that of LiNbO_3_, enabling smaller waveguide bending radii and compact footprints.

In principle, ferroelectric-material-based devices are able to possess ultra-fast response times, reaching the picosecond or even sub-picosecond range. However, to date, only LiNbO_3_-based modulators have demonstrated bandwidths of over 100 GHz. One of the main limitations is the physical properties of fabricated thin films. There is a discrepancy between the actual EO coefficient and the refractive index of grown thin films compared to the values reported for bulk materials, leading to a mismatch between the optical and the microwave velocities that degrade the EO bandwidth, particularly for BaTiO_3_, which has a relatively high dielectric constant at RF frequencies.

Nonetheless, the high production costs and the challenges associated with scaling up to 8-inch wafer manufacturing remain significant barriers for the extensive application of ferroelectric-material-based platforms. Among the four platforms, only LNOI has achieved a commercial product at the 8-inch wafer scale, albeit at a relatively high cost. For LTOI and PZT, researchers have reported wafer-scale manufacturing. BaTiO_3_ has yet to achieve mature wafer-level thin-film manufacturing technology on a PIC-compatible platform. The high chemical inertness of LiNbO_3_ and BaTiO_3_ also contributes to increased costs related to etching processes in production.

In addition, limited by the large bandgap, ferroelectric materials are not ideal candidates for photodetection at communication wavelengths. Therefore, it is critical to investigate photodetection schemes on ferroelectric platforms. This is essential not only for receivers but also for the potential on-chip power monitoring of transmitters, such as bias point monitoring for MZMs and wavelength-locking for microring modulators.

## 4. Conclusions and Perspectives

In the past decade, outstanding performance and innovative functionalities have been reported for Pockels effect-based modulators. In this review, we present a comprehensive summary of the working principle, fabrication techniques, integration schemes, and latest breakthroughs for integrated modulators based on four ferroelectric materials, namely, LiNbO_3_, BaTiO_3_, PZT, and LaTiO_3_.

Leveraging their exceptional electro-optic properties, Pockels effect-based modulators represent a promising frontier for achieving potentially outstanding bandwidth, power efficiency, and compactness in next-generation communication systems. The matured LNOI platform has enabled the extensive development of LiNbO_3_-based modulators with demonstrated ultra-high bandwidth and diverse schemes to optimize the modulation efficiency and footprints. With a compelling EO coefficient, BaTiO_3_-based modulators have emerged in the last few years, achieving superior modulation efficiency. Additionally, PZT and LiTaO_3_ are also promising candidates due to their ease of fabrication and low optical birefringence, respectively.

Furthermore, the advantages of the Pockels effect-based modulators extend their application beyond data communication. There is already research evaluating the performance of Pockels effect-based modulators in neuromorphic photonic processors [111] and quantum photonics [112,113]. In addition, they also hold promising potential in diverse technology fields requiring high modulator functionalities, including sensing [114], satellite data links [115,116], and beyond. In conclusion, as research continues to push the boundaries of material science and manufacturing technology, ferroelectric-material-based modulators are poised to emerge as competitive candidates for future advanced photonics integration systems.

## Figures and Tables

**Table 1:** Physical properties of ferroelectric materials.

Material	Point Group	EO Coefficient (pm/V)	Refractive Index	Curie Temp. (°C)	Reference
LiNbO_3_	3 m	$r_{13}$ = 9.6 $r_{22}$ = 6.8 $r_{33}$ = 30.9 $r_{42}$ = 32.6	$n_{o}$ = 2.286 $n_{e}$ = 2.2	1140	[41,42]
BaTiO_3_	4 mm	$r_{13}$ = 8 $r_{33}$ = 28 $r_{51}$ = 800	$n_{o}$ = 2.444 $n_{e}$ = 2.383	120	[43]
PZT	4 mm	$r_{c(001)}$ = 270.2 $r_{c(011)}$ = 198.2 $r_{c(111)}$ = 125.3	$n_{o}$ = 2.453 $n_{e}$ = 2.458	340	[44,45,46]
LiTaO_3_	3 m	$r_{33}$ = 30.5	$n_{o}$ = 2.119 $n_{e}$ = 2.123	610–700	[47]

**Table 3:** State of the art of BaTiO_3_-integrated modulators.

Year	Structure	V_π_L (V·cm)	Bandwidth (GHz)	Insertion Loss (dB)	Ref.
2024	MRM	1.881	24	NA *	[98]
2023	MZM	2.32	NA	NA	[91]
2023	MZM	0.48	NA	2	[27]
2023	Plasmonic	0.0144	>70	20.5	[101]
2023	MZM	0.48	262	NA	[105]
2022	Plasmonic	NA	66.43	3	[106]
2021	MZM	0.421	NA	NA	[90]
2020	MRM	NA	30	NA	[107]
2019	MZM	0.23	2	NA	[102]
2017	Photonic crystal	0.66	40	12	[26]

* NA: Not available.

## Data Availability

Data will be available based on request.
